# Preparation, Characterization, Dielectric Properties, and AC Conductivity of Chitosan Stabilized Metallic Oxides CoO and SrO: Experiments and Tight Binding Calculations

**DOI:** 10.3390/polym15204132

**Published:** 2023-10-18

**Authors:** Azza Abou Elfadl, Ali H. Bashal, Talaat H. Habeeb, Mohammed A. H. Khalafalla, Nazeeha S. Alkayal, Khaled D. Khalil

**Affiliations:** 1 Department of Physics, Faculty of Science, Fayoum University, Fayoum 63514, Egypt; azzaaboelfadl@yahoo.com; 2 Department of Chemistry, Faculty of Science, Taibah University, Yanbu 46423, Saudi Arabia; abishil@taibahu.edu.sa; 3 Department of Biology, Faculty of Science, Taibah University, Yanbu 46423, Saudi Arabia; habeeb@taibahu.edu.sa; 4 Department of Physics, Faculty of Science, Taibah University-Yanbu Branch, Yanbu 46423, Saudi Arabia; mkhalfalla@taibahu.edu.sa; 5 Chemistry Department, Faculty of Science, King Abdulaziz University, P.O. Box 80203, Jeddah 21589, Saudi Arabia; nalkayal@kau.edu.sa; 6 Department of Chemistry, Faculty of Science, Cairo University, Giza 12613, Egypt

**Keywords:** chitosan, cobalt (II) oxide, strontium oxide, nanocomposite film, optical properties, dielectric properties, tight-binding calculations, HOMO and LUMO

## Abstract

Polymeric films made from chitosan (CS) doped with metal oxide (MO = cobalt (II) oxide and strontium oxide) nanoparticles at different concentrations (5, 10, 15, and 20% wt. MO/CS) were fabricated with the solution cast method. FTIR, SEM, and XRD spectra were used to study the structural features of those nanocomposite films. The FTIR spectra of chitosan showed the main characteristic peaks that are usually present, but they were shifted considerably by the chemical interaction with metal oxides. FTIR analysis of the hybrid chitosan-CoO nanocomposite exhibited notable peaks at 558 and 681 cm^−1^. Conversely, the FTIR analysis of the chitosan-SrO composite displayed peaks at 733.23 cm^−1^, 810.10 cm^−1^, and 856.39 cm^−1^, which can be attributed to the bending vibrations of Co-O and Sr-O bonds, respectively. In addition, the SEM graphs showed a noticeable morphological change on the surface of chitosan, which may be due to surface adsorption with metal oxide nanoparticles. The XRD pattern also revealed a clear change in the crystallinity of chitosan when it is in contact with metal oxide nanoparticles. The presence of characteristic signals for cobalt (Co) and strontium (Sr) are clearly shown in the EDX examinations, providing convincing evidence for their incorporation into the chitosan matrix. Moreover, the stability of the nanoparticle-chitosan coordinated bonding was verified from the accurate and broadly parametrized semi-empirical tight-binding quantum chemistry calculation. This leads to the determination of the structures’ chemical hardness as estimated from the frontier’s orbital calculations. We characterized the dielectric properties in terms of the real and imaginary dielectric permittivity as a function of frequency. Dielectric findings reveal the existence of extensive interactions of CoO and SrO, more pronounced for SrO, with the functional groups of CS through coordination bonding. This induces the charge transfer of the complexes between CoO and SrO and the CS chains and a decrease in the amount of the crystalline phase, as verified from the XRD patterns.

## 1. Introduction

In nanotechnology, the impact of particle size on materials’ abilities to catalyze reactions is crucial for scientific and industrial applications [1,2]. Nanomaterials exhibit several intriguing properties, including superior mechanical performance, surface functionalization, a high surface area, and controllable porosity in comparison to bulk materials [3]. The type of support being used has an unquestionable significant impact on how supported particles are controlled in terms of size. To create smaller NPs directly on the support, it is desirable to use materials with fewer pores, higher surface areas, and favorable interactions with the particles. These nanotechnology initiatives have been used by the electrical industries to develop applications for its parts, such as electrical transformers, which are important components in the electricity network [4,5]. Chitosan (CS) is a biopolymer derived from chitin, a biodegradable polysaccharide, through the process of alkali deacetylation. Crab shells are inherently found in diverse sources [6]. In recent times, chitosan has attracted much attention as a highly promising substance for a wide range of uses. The primary reason for its widespread use can be attributed to its attractive characteristics, such as its capacity to alter electrode surfaces, its exceptional ability to form films, notable stability, high permeability to water, strong adhesion to electrode surfaces, biocompatibility, lack of toxicity, high mechanical durability, affordability, and amenability to chemical modifications. The aforementioned characteristics are derived from the existence of reactive amino and hydroxyl functional groups within its molecular composition [7]. Chitosan and its derivatives are widely seen as highly efficient templates for synthesizing metal oxide nanoparticles. This is attributed to their unique ability to interact with metal ions through the hydroxyl and amino functional groups [8]. Previous research has shown that the incorporation of various elements, such as metal nanoparticles and carbon nanotubes, into CS films can effectively improve their electrical conductivity [9]. To fulfill the specifications of miniaturized microelectronic device structures, such as gate dielectrics, high charge storage capacitors, and electroactive material, it is imperative to utilize dielectric materials that exhibit high dielectric permittivity and dielectric field strength, while minimizing dielectric loss [10]. Both temperature and frequency exert an influence on the dielectric characteristics of the sample. Dielectric materials have a limited number of free charges when exposed to lower temperatures. However, when the temperature rises, there is an observable rise in the number of carriers that are able to exceed the barrier height. As a result, the exposure of carriers to an external electric field gives rise to the appearance of excessive capacitance and conductance [11]. Chitosan has been employed as a support material for cobalt oxide (CoO) in several applications, including the enhancement of photocatalytic hydrogen evolution [12], the quick colorimetric detection of L-cysteine, and the photocatalytic reduction of hexavalent chromium (Cr(VI)) ions [13].

In a number of recent studies, it has been demonstrated that the presence of particles with a nanometric size enhances the dielectric characteristics of polymers used as electrical insulators (polyesterimide, polyethylene, epoxy resins, polyimide, and polyamide) [14,15,16,17,18]. Metal oxide nanoparticles are incorporated directly into the polymer matrix during the in situ synthesis of polymer–metal oxide nanocomposite materials. With these techniques, it has been possible to disperse nanoparticles uniformly and strengthen the interfacial bonds between the nanoparticles and the polymer matrix. Sol-gel processing methods [19], electrospinning [20], and solution casting [21] are a few of the popular in situ synthesis procedures.

**Figure 1:**
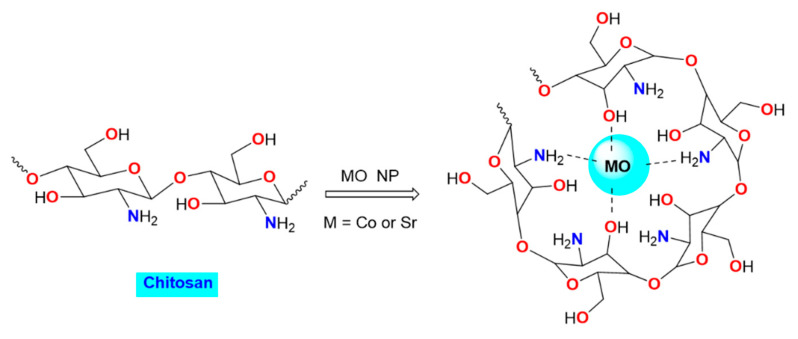
The chitosan-metal oxide (CoO or SrO) nanocomposite in a simplified viewpoint.

CoO and Co_3_O_4_ are the two stable oxides formed by cobalt. Cobalt oxide nanoparticles can be manufactured through homogeneous precipitation under a variety of synthetic conditions [22]. On a variety of substrates, cobalt oxide thin films have been created using a range of deposition methods, including the sol-gel process, spray pyrolysis, chemical vapor deposition, electrophoretic deposition, etc. [22,23]. Cobalt oxide nanoparticles can be used in various applications including micro-electronics, micro-batteries, electrode active materials, superconductors, electronic ceramics, and temperature and gas sensors [24]. In contrast, the distinctive characteristics of nanoparticles composed of strontium oxide have garnered significant interest in both fundamental and practical investigations pertaining to the development of electronic devices [25,26]. Due to its exceptional thermal stability, this substance is utilized in several industries such as ceramics, glass, and optics [27,28]. Strontium oxide nanoparticles exhibit morphological stability, rendering them suitable for a range of applications including dye-sensitized solar cells, lithium-ion battery electrodes, transistors, semiconductors, supercapacitors, solar cells, and gas sensors [29]. Chitosan-strontium oxide also exhibits catalytic efficiency. A recent study [30] has demonstrated that the utilization of chitosan-strontium oxide nanocomposites enables the synthesis of 1,3,4-thiadiazoles. These compounds possess several potential applications such as antibacterial, antifungal, antihepatitic, antioxidant, and others [31]. Sol-gel synthesis is one of the several techniques used to create these nanomaterials in the liquid phase, including hydrothermal, micro-emulsion, sol-gel, and microbial processes [32]. Sol-gel synthesis is straightforward and reasonably priced [33]. For the aforementioned reasons, herein we tried to prepare chitosan stabilized metallic oxides, chitosan/CoO, and chitosan/SrO nanocomposites (Figure 1), and studied the structural properties of these hybrid materials and investigated their electrical properties to fit many related applications. Some of the probable future applications that could benefit from the findings of our work include the super capacitor [34], Lithium-ion batteries [35], and biodiesel [36] applications. Another interesting application that is closely relevant to our present work is the use of chitosan as a nano-delivery system for cancer therapy [37]. For instance, our work has demonstrated the stability of the CoO nanoparticle on the Chitosan, a finding that has a direct impact on the recently reported “Biomimetic CoO@ AuPt nanozyme responsive to multiple tumor microenvironmental clues for augmenting chemodynamic therapy” [38].

## 2. Methodology

### 2.1. Materials and Instruments

In the present study, chitosan (medium molecular weight; ≥75% deacetylated chitin; shrimp shells) powder was purchased from Sigma Aldrich company (batch C3646, density = 0.15–0.3 g/cm^3^). Cobalt (II) oxide (CoO, powder, product no. 529443, purity 99.99% based on trace metals analysis, <150.0 ppm) and strontium oxide (SrO, powder, product no. 415138) were used. Other chemicals, such as potassium hydroxide, methanol, acetic acid, and water, were purchased from Merck Company and were used as such without any further purification. A Nicolet Magna 6700 FT spectrometer (Thermo Fisher Scientific, Waltham, MA, USA) was used to record Fourier transform infrared spectra in the wavenumber range (500–4000 cm^−1^). A Philips diffractometer (Model: X’Pert-Pro MPD; Philips, now PANaytical, Malvern, Worcestershire, UK) with Cu Kα radiation (wavelength 1.5418 Å) at 40 kV and 40 mA X-ray diffraction (XRD) patterns were used, and the patterns were recorded in 2θ range from 5° and 60°, with 1.5 degree/min scan speed. The thin films were cut into small pieces and attached to the SEM stubs with carbon tape for SEM and EDX measurements (HRSEM, JSM 6510A, Jeol Ltd., Tokyo, Japan). Samples were coated with a 4 nm thick platinum layer and then transferred to the SEM Teneo/Quattro for imaging. The impedance measurements of samples were carried out at temperatures range 298 to 393 K by a Solartron Analytical, Frequency Response Analyzer (FRA). The spectra were recorded in the frequency range 1 k Hz to 1000 kHz. The complex impedance (Z*) was transformed to the complex permittivity (ε*) using the equation: ε* = 1/jωC_o_Z*, where ω and *Co* are the angular frequency and the vacuum capacitance, respectively.

For the electrochemical calculation, we used the tight-binding semi-empirical quantum chemistry, XTB (Skyliner, Poland) [39], software for the optimized geometries of CoO/chitosan and SrO/chitosan (structure of CoO and SrO nano-clusters attached to the chitosan as shown later in Section 3.3), and electronic properties calculations. The Mol-Instincts website allows users to download the three-dimensional (3D) structure of chitosan C_56_H_103_N_9_O_39_. The CoO and SrO nano-clusters were then attached to the central part of the chitosan, followed by optimization runs to search for the best site for the coordination bonding between the molecule and the chitosan, thanks to the energy minimization search algorithm of xtb. For the first principle calculation, we have calculated the frontier molecular orbitals: the highest occupied molecular orbital (HOMO) and the lowest unoccupied molecular orbital (LUMO). To visualize these orbitals around the molecular structure we employed the Multiwfn software [40] by setting the HOMO and LUMO isosurfaces at −0.02 au (green isosurface) and 0.02 au (blue isosurface). The sign of the orbital level indicates the wave function polarity.

### 2.2. Preparation of CS-CoO and CS-SrO Nanocomposite Films

**Figure 2:**
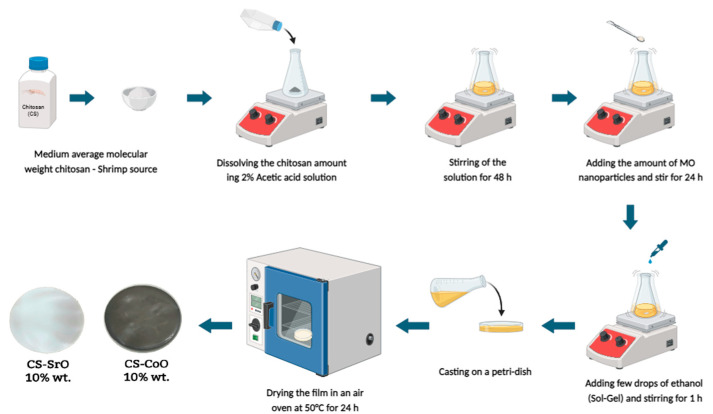
Sol-gel preparation of chitosan-metal oxide (CoO or SrO) nanocomposite.

The synthesis of the chitosan-metal oxide (CS/CoO or CS/SrO) nanocomposite films was carried out using the sol-gel approach [20,41]. A 2% weight chitosan solution was created by dissolving 1 g of chitosan in 50 milliliters of a 2% weight/volume aqueous acetic acid solution. The dissolution process involved continuous stirring at room temperature for a duration of 48 h. The viscous solution was filtered using a 90 mm Whatman filter paper in order to obtain a homogeneous and transparent chitosan solution. A fraction of the solution was transferred into a 50 mL vial, where 5 (*w*/*v*%) quantities of metal oxide (either CoO or SrO) were incrementally added. The resulting combination was then violently agitated for an extra duration of 24 h. A precise volume of ethanol (0.5 mL) was introduced into the system to facilitate the sol-gel process while being subjected to continuous stirring for an additional duration of 1 h. In order to facilitate the evaporation of the solvent, the solution was introduced into a Teflon Petri dish with a diameter of 8 cm. Subsequently, the solution was subjected to a drying process for a duration of three days within a vacuum oven operating at a temperature of 50 °C. Following the neutralization process with 5 mL of 1 M KOH and subsequent removal from the Petri plate, the chitosan-MO nanocomposite film was immersed in distilled water. The film was thereafter placed in a vacuum desiccator at room temperature for a duration of two days. Figure 2 shows a block drawing for the sample preparation.

## 3. Results and Discussion

### 3.1. Characterization of the Nanocomposite Film Made of Chitosan and Metal Oxides

#### 3.1.1. FTIR Characterization

**Figure 3:**
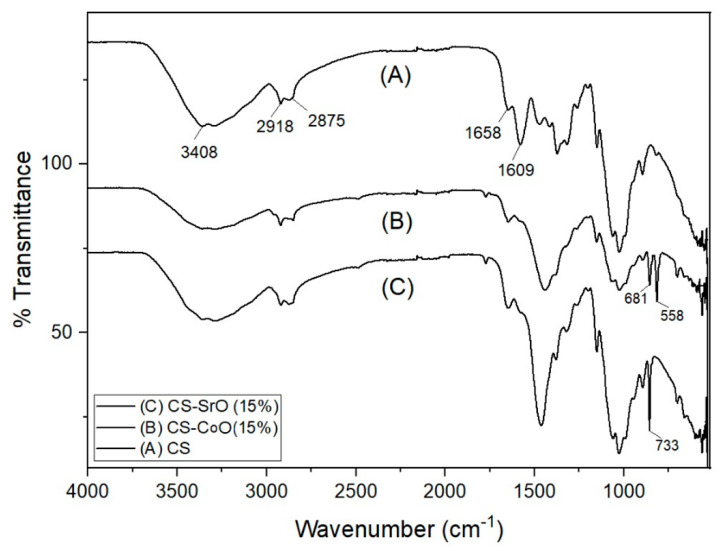
FTIR spectra of chitosan (A), chitosan-cobalt oxide nanocomposite (B) (5 wt.%), and chitosan-strontium oxide nanocomposite (C) (5 wt.%) are shown side by side.

In Figure 3, a comparative FTIR analysis of native chitosan (A), chitosan-cobalt oxide (B), and chitosan-strontium oxide (C) nanocomposite is shown. Due to the overlapped stretching bands for -OH and -NH_2,_ that are located in the same region, the chitosan spectrum (A) revealed a large stretching band at υ = 3408 cm^−1^ [41,42,43]. Additionally, the typical chitosan characteristic bands (amide group band for CONH) developed at υ = 1658 and 1609 cm^−1^, and those of aliphatic CH emerged at υ = 2918 and 2875 cm^−1^.

Figure 3B displays the FTIR of the hybrid Chitosan-CoO nanocomposite, which revealed significant alterations, particularly in the small wavenumber region. The CoO NPs have two distinct peaks at 558 and 681 cm^−1^ that are documented in the literature and can be intimately associated with Co-O bending vibrations [44,45]. The hybrid chitosan-SrO nanocomposite, however, is depicted in Figure 3C, and there are obvious changes in the small wavenumber region. This is consistent with what has been written in the literature, which states that the SrO NPs have peaks at 733 cm^−1^, 810 cm^−1^, and 856 cm^−1^ that are attributed to the Sr-O bending vibrations [29]. Additionally, the existence of an additional peak at 854 cm^−1^ is seen as unmistakable proof that strontium oxide was included and coordinated with the binding sites along the chitosan backbone.

#### 3.1.2. SEM and Morphological Characteristics

**Figure 4:**
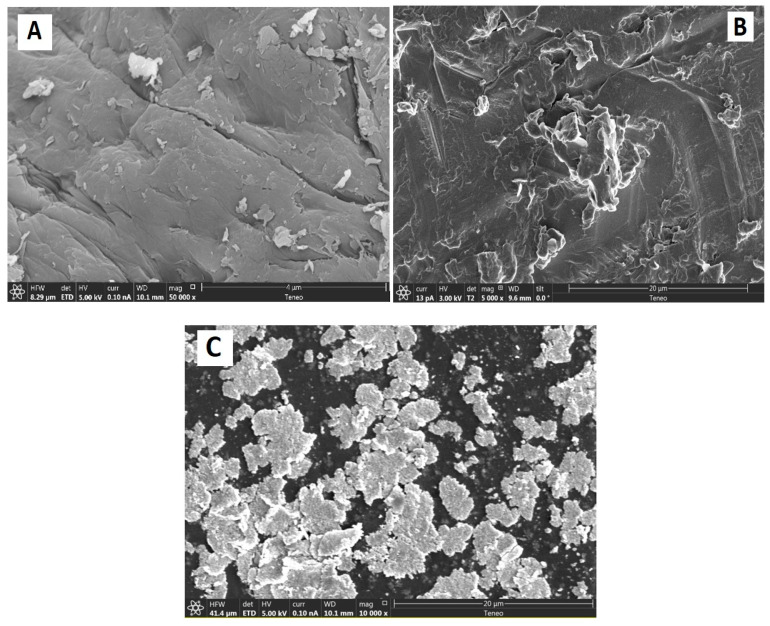
FESEM of chitosan (**A**), chitosan-CoO nanocomposite, 5 wt.% (**B**), and chitosan-SrO composite, 5 wt.% (**C**).

Figure 4 displays FESEM images of chitosan and chitosan that has been treated with CoO and SrO nanoparticles. The morphological alterations to the surface of the chitosan caused by its interaction with the metal oxide molecules are visible in the SEM pictures.

The unaltered chitosan (A) micrograph showed a typical non-porous, fibrous-like surface in conformity with what has previously been described in the literature [41,42,43]. Image 3B shows a clear change with the incorporation of cobalt oxide on the chitosan surface as compared to the surface of native chitosan. The image of the SrO nanoparticles in Figure 4C demonstrates their spherical shape and some aggregations, as described in the literature [29]. The CS-SrO nanocomposite (C) then demonstrated clear distributions of aggregations, which are rationalized by the interaction of SrO molecules in these polymer areas.

#### 3.1.3. Estimating the Quantity of Cobalt and Strontium in the Polymer Matrix Using Energy-Dispersive X-ray Spectroscopy (EDS)

**Figure 5:**
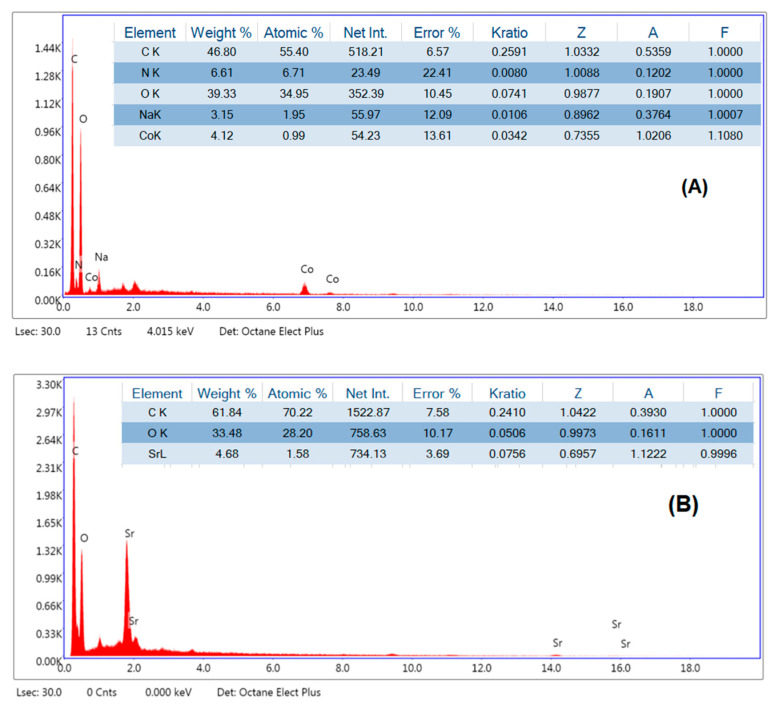
Energy Dispersive Spectroscopy of chitosan-CoO (**A**) (5 wt.%), and chitosan-SrO nanocomposites (**B**) (5 wt.%).

In order to estimate the amount of metal in the chitosan, Figure 5A exhibits the EDS of the chitosan-CoO nanocomposites, where the hybrid material’s EDS confirmed the appearance of the standard Co signals, and the Co content was 4.12 wt.%, as seen in Figure 5. The chitosan-SrO nanocomposites’ EDS graph in Figure 5B displays the appearance of typical Sr signals; the materials’ Sr concentration was 4.68 weight percent. The existence of the typical Co and Sr signals is evident from the EDX tests, which strongly supports their inclusion into the chitosan matrix.

#### 3.1.4. X-ray Diffraction

**Figure 6:**
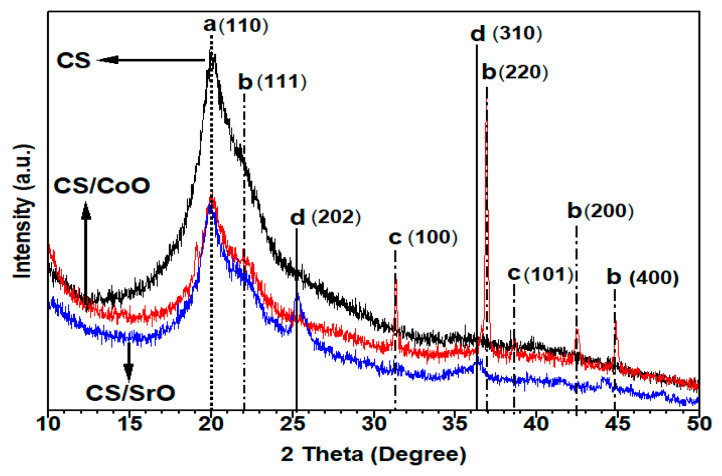
XRD of CS (a), CS-CoO (5 wt.%) (b), and CS-SrO (5 wt.%) (d) nanocomposites. Note, (c) diffraction peaks related to β-Co(OH)_2_.

X-ray diffraction (XRD) has been employed as a technique to investigate the level of crystallinity and the nanostructural characteristics of both the pristine chitosan material and the thin films composed of modified chitosan incorporating 5 wt.% CoO and 5 wt.% SrO nanoparticles. The XRD pattern of unmodified chitosan, as depicted in Figure 6, displays a distinct diffraction peak (a) at an angle of 20.8°. This peak is ascribed to the presence of (110) crystallographic planes inside the chitosan structure [46,47]. The existence of both crystalline and amorphous components of chitosan is indicated by this diffraction peak, as reported in the literature [41,42,43]. Moreover, the presence of certain chitosan impurities was revealed by the appearance of supplementary peaks in the X-ray diffraction (XRD) pattern. The characteristic peak of chitosan (a) was observed in the diffraction patterns of both the CS-CoO and CS-SrO nanocomposites at an identical angle of 20°. The absence of any shift in the peak position between undoped chitosan and doped chitosan suggests that the cubic CoO and SrO nanocrystals that were synthesized do not exhibit any additional strain. This observation was also noted by Deori and Deka [48]. Furthermore, the XRD technique was employed to ascertain the crystal structure of the synthesized CS-CoO. The diffractogram is also depicted in Figure 6. The CoO nanoparticles exhibit distinct peaks at 2θ = 22.03°, 36.80°, 42.46°, and 44.9° corresponding to the crystallographic planes (111), (220), (200), and (400) as shown on the JCPDS card no. 73-1701 [48]. The XRD analysis indicated the existence of CoO crystals within the produced nanoparticles. Nevertheless, an alternative phase, specifically β-Co(OH)_2_ (JCPDS card #30-443), has been seen at angles of 2θ = 31.4°and 36.80°, denoted by their corresponding Miller indices, (100) and (101) [49]. This finding is consistent with a prior study conducted by Sun et al. [50]. Furthermore, the absence of impurity diffraction peaks suggests that the Co-precursor has undergone complete transformation into its oxide forms. The XRD pattern did not exhibit the presence of any further phases, such as Co_3_O_4_ (JCPDS card #42-1467) or metallic cobalt (Co, JCPDS #05-0727). The nanocomposite film, consisting of a crystal phase produced with 5 wt.% CS-SrO, was successfully detected using X-ray diffraction measurement. The XRD pattern of strontium oxide nanoparticles is also illustrated in Figure 6. The diffraction peaks exhibit a high level of concordance with the standard data for strontium oxide as documented in the JCPDS card No. 01-1113. The Bragg peaks are seen at the angles of 2θ = 25.19° and 36.37°, corresponding to the crystallographic indices (202) and (310), respectively [51].

### 3.2. Dielectric Properties

**Figure 7:**
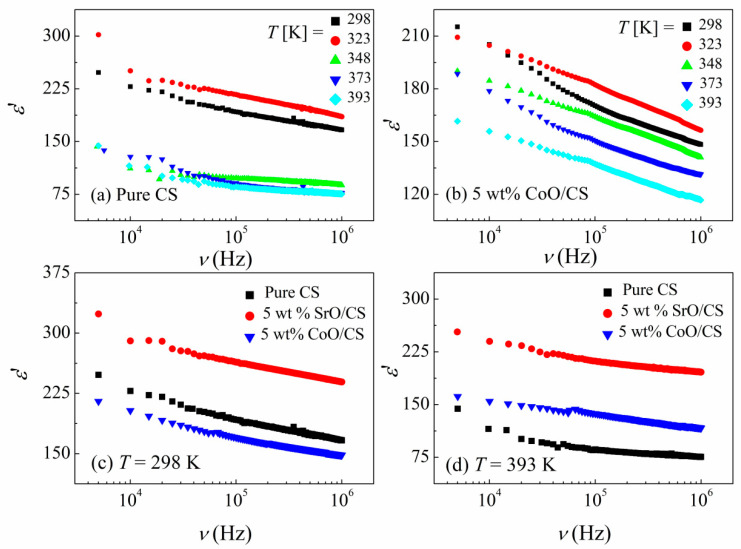
Frequency dependences of the real part of permittivity, ε′ (ν), at selected temperatures for (**a**) CS, (**b**) CoO/CS nanocomposite, (**c**) all samples at 298 K, (**d**) all samples at 393 K.

The dielectric response to the applied electric field can be due to several processes such as the relaxation processes from the molecular fluctuations of dipoles, the conduction process from the propagation of charge carriers, and the interfacial (or Maxwell/Wagner/Sillars (MWS)) polarization in multiphase systems due to the blocking of charge carriers at the interfaces of the different phases [52]. In the present paper, we characterized the dielectric properties in terms of the real (ε′), named dielectric constant, and the imaginary (ε″), named dielectric loss, parts of the complex dielectric permittivity ε* as a function of frequency to explore the effect of CoO and SrO nanoparticles on the dipolar relaxation and the electrical conduction processes of CS for electrical applications, e.g., in energy storage devices. Figure 6a,b, shows the dielectric constant as a function of frequency, ε′ (υ), at various temperatures for pure CS and CoO/CS samples, while Figure 7c,d illustrates the ε′ (υ) spectra for CS, CoO/CS, and SrO/CS nanocomposites at 298 K and 393 K, respectively.

For all samples, the ε′ (υ) spectra depend on the frequency as well as temperature. Each sample exhibits a significant decrease in the ε′ values with increasing frequencies. The continuous decrease in ε′ with increasing frequency is quite common in all dielectric materials. When the frequency increases, the polarization mechanisms decrease due to the reduced number of dipoles aligned in the applied field’s direction.

At high frequencies, the dipoles in the macromolecules lag the alternation of the electric field, therefore their contribution to ε′ will be lowered. However, at low frequencies, the matching between the dipole orientation and the frequency change speeds increases the number of participating dipoles in polarization. In addition, the ε′ spectrum depends significantly on the temperature. The ε′ value initially increased with temperature, attaining a maximum at a temperature of 323 K for CS and nanocomposites. A further increase in temperature reduces the ε′ value and remains almost constant at elevated temperatures ≥363 K for pure CS. Similar results were reported for CS by El-Sayed et al. [53].

The viscosity of materials decreases with temperature and the absorbed thermal energy allows the dipoles to orient and follow the external field, consequently, enhancing the ε′ value. Moreover, the growth in ε′ with temperature could be due to the disentanglements of the molecular chains, which are eased by molecular vibrations [54]. Furthermore, it is evident that the temperature dependence becomes stronger when adding CoO and SrO into CS.

As shown in Figure 7c,d, at a high *T*, the ε′ value of the CS is enhanced with the addition of CoO and SrO, which could be attributed to interfacial polarization resulting from the dielectric performance difference between the CS and the metal oxides. The SrO/CS nanocomposite exhibits the maximum ε′ when compared to the others. However, at lower temperatures (*T* ≤ 323), the CoO/CS nanocomposite shows ε′ values less than those of CS which could reflect the microstructure change of the nanocomposite with heating.

Dielectric relaxations and electrical conductivity are responsible for the dielectric losses in dielectrics. The temperature dependence of the ε″ suggests the nature of the observed losses in the materials. The dielectric loss originating from the dielectric relaxation would decrease with increasing temperatures, whereas that originating from the electrical conductivity would decrease with decreasing temperatures.

**Figure 8:**
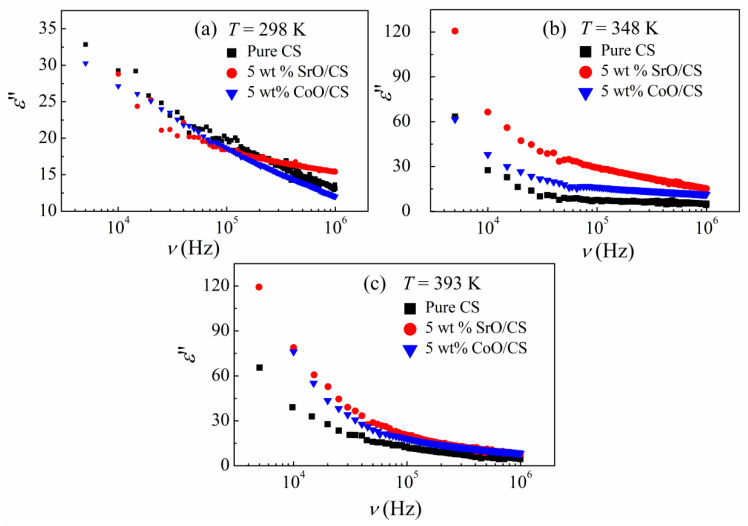
Frequency dependencies of the imaginary part of permittivity, ε″(ν), at temperatures (**a**) 298 K, (**b**) 348 K, and (**c**) 393 K for CS and its (CoO, SrO) nanocomposites.

Figure 8 presents the dielectric loss spectra, ε″(ν), of pure CS, CoO, and SrO/CS nanocomposites at different temperatures. For each sample, no relaxation peak is identified within the considered range of the temperature and frequency, however, the ε″ value rises sharply at low frequencies evincing the dc conduction and interfacial process contributions [55,56]. Furthermore, it has been shown that the elevation of the temperature to 323 K results in an increase in the value of ε″, indicating that the loss is derived from electrical conductivity. Nevertheless, when the temperature continues to rise, the values of ε″ decrease further, indicating that the losses seen are mostly influenced by the dielectric relaxations occurring at elevated temperatures.

An inspection of Figure 8 indicates that CS nanocomposites possess dielectric losses lower than those of pure CS at 298 K. However, at high temperatures, the ε″ spectra reveal an increase with the addition of CoO and SrO, which suggests that the addition of a metal oxide strengthens the interfacial polarization effect of the nanocomposites. Similar behavior was observed for CS based nanocomposites [57,58]. Neagu et al. investigated the electric response of CS and BaTiO_3_/CS composites, with different BaTiO_3_ contents, at room temperature. The permittivity showed an increment together with a reduction in the dielectric losses with increasing BaTiO_3_ content. Similarly, Alshammari found that the ac conductivity, ε′, and ε″ of CS were increased with increasing concentrations of ZnO/GO nanofiller in the films.

Bhatt et al. studied the effect of Co_3_O_4_ fillers on the dielectric properties of CS in the temperature range 303–343 K and reported that the permittivity and conductivity of CS were increased with increasing Co_3_O_4_ content [59]. To verify the origin of the loss in our samples, we compared the ε″ spectra at different temperatures, cf. Figure 8. The spectra of CS and its CoO and SrO nanocomposites display a strong temperature dependence. At *T* ≤ 323 K, the increment of ε″ with increasing *T* suggests that the loss originates from the electrical conductivity, whereas the decreasing trend of ε″ at temperature of *T* > 323, suggests that the observed losses are dominated by the dielectric relaxations. According to the data presented in Figure 7 and Figure 8, it can be observed that the SrO/CS nanocomposite exhibits the lowest dielectric loss and the highest dielectric constant when subjected to low temperatures and frequencies. This characteristic renders it suitable for a wide range of applications, including its use as embedded and integral thin film capacitors, as well as in high dielectric constant layers within transistors. In recent times, there has been a notable focus on capacitors due to their wide range of applications, including, but not limited to, filtering, decoupling, bypassing, and timing functions. In addition to ε*, the response of materials to alternating electric fields (sinusoidal fields) is commonly represented in terms of the complex ac conductivity s* = i *ω ε*_o_ ε* to understand the conduction mechanism for electrical applications, e.g., in energy storage devices.

**Figure 9:**
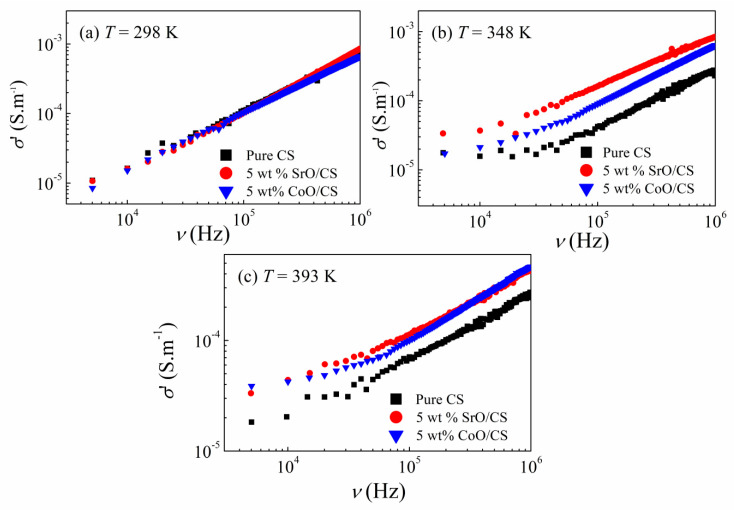
The variation of σ′ with frequency for CS (**a**) and its CoO (**b**) and SrO (**c**) nanocomposites at different temperatures.

The real part of the complex ac conductivity, σ′ (ν), was calculated using the relation: σ′ = ε″ εₒ ω, where εₒ is the permittivity of free space and ω is the angular frequency (ω = 2πν). Figure 9 depicts the frequency response of the conductivity, σ′ (ν), for the pure CS and CoO, SrO/CS nanocomposites at different temperatures.

It is evident that the σ′ spectra display two different regions at high temperature, demonstrating the presence of various dissipated effects, the almost frequency-independent region, and a power-law dependence on frequency at low and high frequency ranges, respectively. The first region reflects the DC contribution arising from the long-range charge transport, whereas the second region signifies the existence of intrinsic relaxation. Generally, conductivity spectra of various materials are investigated via Jonschers universal power low [60,61]; σ′ = σ_dc_ + *A* ω^m^, where σ_dc_ is DC conductivity, A is temperature-dependent constant and m is an exponent reflecting the interaction degree between the transferring charge entities and the surrounding lattice.

The variation in the exponent m with temperature specifies the ac conduction mechanism. From the slope of ln σ versus ln ω, Figure not shown, the exponent m is estimated in the high frequency range at different temperatures for all samples. As the temperature increases from 298 K to 393 K, the exponent m decreases from 0.82 to 0.58, from 0.81 to 0.68, and from 0.92 to 0.57 for pure CS, CoO/CS, and SrO/CS, respectively. The decreasing trend of the exponent with temperature evinces that the correlated barrier hopping model (the CBH model) is the dominant conduction mechanism of the charge carriers in our samples [62,63].

Compared to CS, CoO/CS and SrO/CS nanocomposites have a higher conductivity, which could be ascribed to the reduction in the crystallinity of CS, consequently, decreasing the energy barrier and assisting the charge transport, cf. XRD results. Based on the free volume theory [64], molecular mobility depends on the free volume. The free volume in the amorphous phase is higher than in the crystalline phase, therefore, the ionic mobility increases when increasing the amorphous phase resulting in higher ionic conductivity [65,66]. Moreover, the addition of CoO and SrO could form new energy levels within the band gap of CS which act as traps for the hopping charge carriers, hence enhancing the conductivity of CS nanocomposites.

In conclusion, dielectric findings reveal the existence of extensive interactions of CoO and SrO, which are more pronounced for SrO, with the functional groups of CS through the coordination bonding that induces the existence of charge–transfer complexes between CoO and SrO and the CS chains, and the decrease in the amount of the crystalline phase, as noticed in the XRD patterns.

In their study, Rahman et al. [67] observed that the incorporation of nano ZnO resulted in enhancements in both the dielectric constant and ac-conductivity of the CS film. In their study, Ibrahim et al. [68] observed a notable rise in the dielectric constant of the chitosan–polyethylene oxide (Ch. (80%)-PEO (20%)) blend following the introduction of graphene oxide (GO) and multi-walled carbon nanotubes (MWCNTs). Additionally, the nanocomposites exhibited low dielectric loss values, indicating their potential suitability for energy storage applications. Furthermore, the nanocomposites composed of MWCNTs, GO, and a blend material have alternating current (AC) conductivity that is four times greater than that of the pure blend material. The dielectric results indicate the presence of significant contacts between CoO and SrO, with a stronger effect observed for SrO. These interactions are mostly attributed to coordination bonding between the functional groups of CS and the metal oxides. The occurrence of diverse electron-rich polar sites, such as amino (-NH_2_), hydroxyl (-OH), and ether (–O–) functional groups within the CS structure, facilitates the creation of charge–transfer (CT) complexes. This is achieved through the process of charge sharing, wherein nitrogen and oxygen atoms donate their unshared electron pairs to the metal ions. The decrease in the quantity of the crystalline phase of CS may be ascribed to the donor–acceptor interaction between CS and CoO and SrO.

### 3.3. Tight-Binding Calculations of HOMO and LUMO and Other Electrochemical Properties

**Figure 10:**
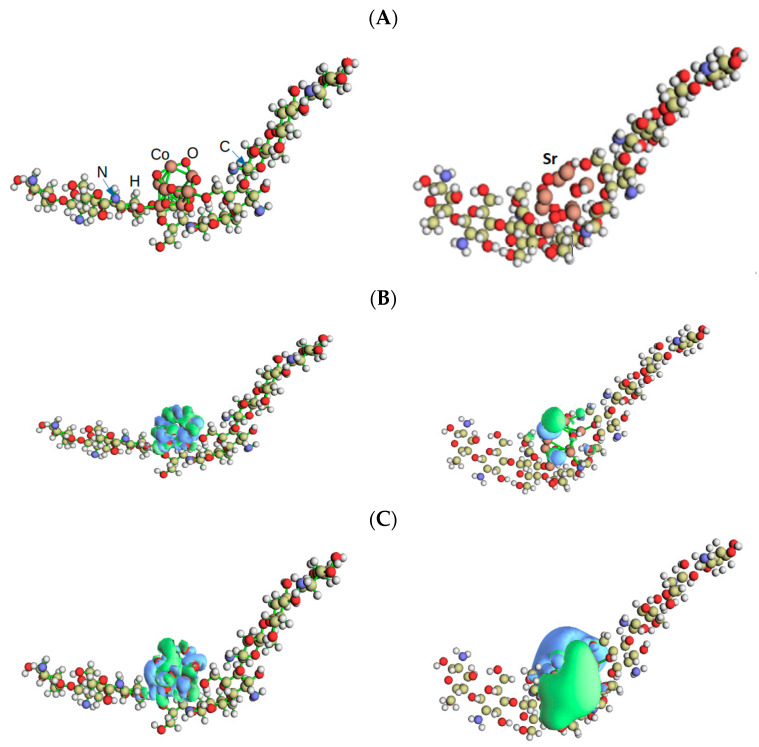
(**A**) The geometrically optimized structure of the CoO/chitosan (left panel, CoO/C_56_H_103_N_9_O_39_) and SrO/chitosan, right panel, the calculated HOMO isosurface (where the signs of the wavefunction are “+” and “–” for the blue and green, respectively) is shown in (**B**), while the LUMO isosurface is shown in (**C**). The isosurface value is 0.01 au.

The binding energies, *E*_b_ = *E*_combind_ − (*E*_Chitosan_ + *E*_nano_), were estimated as −9.2 eV and −8.1 eV, respectively, for the compound’s CoO/chitosan and SrO/chitosan, where *E*_combind_ is the compound’s total energy, *E*_chitosan_ is the chitosan’s energy, and *E*_nano_ is the energy for the nano-cluster (Co_4_O_4_, or Sr_4_O_4_). The negative *E*_b_ indicates the stability of the compounds. Figure 10A shows the geometrically optimized structure for the compounds where the HOMO (Highest Occupied Molecular Orbital) and LUMO (Lowest Occupied Molecular orbitals) iso-surfaces are shown in Figure 10B,C, respectively. These orbitals are mainly localized around nano-clusters, suggesting the chemical stability of the CoO/chitosan structure. The nearly spherical spreading of the HOMO and LUMO around CoO nano-cluster (left panels of Figure 6b,c) may be associated with the filling of the distributed and orientated components of the *d*-orbitals of the Co transition metal. On the other hand, the HOMO and LUMO exhibit different profiles around the SrO nano-clusters (right panels of Figure 6b,c) which may be due to the Alkaline, s-block nature of the Sr metal.

The chemical hardness η = −(*E*_HOMO_ − *E*_LUMO_)/2 of SrO/CS is −1.1 eV, and that fof CoO/CS is −0.3 eV where *E*_HOMO_ and *E*_LUMO_ are the HOMO and LUMO energies. Chemical hardness is an important descriptor of the structure’s resistance against its deformation since it has been linked to the stability of the chemical system. A reduced hardness will make the molecule more stable and able to withstand changes in its electronic distribution and/or polarization. This suggests that SrO/CS has better chemical stability as compared to CoO/CS. In our future work we will consider the effect of increasing the number of the attached SrO and CoO molecular units on the enhancement of this parameter.

## 4. Conclusions

In this study, 5 wt.% of cobalt (II) oxide and strontium oxide nanoparticles have been inserted into chitosan by applying the simple solution casting approach. The nanocomposite films were characterized by FTIR, FESEM, EDS, and XRD measurements. All the tools’ findings confirmed the metal oxide molecules’ presence in the chitosan matrix. We characterized the dielectric properties in terms of the real (ε′), and the imaginary (ε″) parts of the complex dielectric permittivity ε*) as a function of frequency. Dielectric findings reveal the existence of extensive interactions of CoO and SrO, which are more pronounced for SrO, with the functional groups of CS through the coordination bonding that induces the existence of charge–transfer complexes between CoO and SrO and the CS chains and the decrease in the amount of the crystalline phase, as confirmed with the XRD patterns. Additionally, the stability of the nanoparticle–chitosan coordinated bonding was verified from the accurate and broadly parametrized semi-empirical tight-binding quantum chemistry calculation. This leads to the determination of the structures’ chemical hardness as estimated from the frontier’s orbital calculations.

## Data Availability

Data will be available by request.
